# Regulation of *Nematostella* neural progenitors by SoxB, Notch and bHLH genes

**DOI:** 10.1242/dev.123745

**Published:** 2015-10-01

**Authors:** Gemma Sian Richards, Fabian Rentzsch

**Affiliations:** Sars Centre for Marine Molecular Biology, University of Bergen, Thormøhlensgate 55, Bergen N-5008, Norway

**Keywords:** Cnidaria, Proneural genes, DAPT, Neurogenesis

## Abstract

Notch signalling, SoxB and Group A bHLH ‘proneural’ genes are conserved regulators of the neurogenic program in many bilaterians. However, the ancestry of their functions and interactions is not well understood. We address this question in the sea anemone *Nematostella vectensis*, a representative of the Cnidaria, the sister clade to the Bilateria. It has previously been found that the *SoxB* orthologue *NvSoxB(2)* is expressed in neural progenitor cells (NPCs) in *Nematostella* and promotes the development of both neurons and nematocytes, whereas Notch signalling has been implicated in the negative regulation of neurons and the positive regulation of nematocytes. Here, we clarify the role of Notch by reporting that inhibition of Notch signalling increases the numbers of both neurons and nematocytes, as well as increasing the number of *NvSoxB(2)*-expressing cells. This suggests that Notch restricts neurogenesis by limiting the generation of NPCs. We then characterise *NvAth-like* (Atonal/Neurogenin family) as a positive regulator of neurogenesis that is co-expressed with *NvSoxB(2)* in a subset of dividing NPCs, while we find that *NvAshA* (Achaete-scute family) and *NvSoxB(2)* are co-expressed in non-dividing cells only. Reciprocal knockdown experiments reveal a mutual requirement for *NvSoxB(2)* and *NvAth-like* in neural differentiation; however, the primary expression of each gene is independent of the other. Together, these data demonstrate that Notch signalling and *NvSoxB(2)* regulate *Nematostella* neural progenitors via parallel yet interacting mechanisms; with different aspects of these interactions being shared with *Drosophila* and/or vertebrate neurogenesis.

## Abstract (precis)

**Summary:** In the sea anemone *Nematostella vectensis*, SoxB and Atonal-like genes, which participate in Notch signalling, regulate neural progenitor specification via parallel yet interacting mechanisms.

## INTRODUCTION

Cnidarians (jellyfish, corals, sea anemones) are the sister clade of the Bilateria (Hejnol et al., 2009; Pick et al., 2010), and they possess simple, nerve-net based nervous systems comprising three classes of neural cells – sensory neurons, ganglion neurons (analogous to interneurons) and nematocytes (mechano-/chemoreceptor cells). Due to their relative phylogenetic positions, identifying conserved features of cnidarian and bilaterian neurogenesis can inform reconstructions of the ancestral neurogenic characters of eumetazoans (herein referring to Bilateria+Cnidaria). Indeed, genomic comparisons have shown that cnidarians possess many orthologues to key bilaterian neural-related genes (Chapman et al., 2010; Galliot et al., 2009; Putnam et al., 2007; Watanabe et al., 2009), but a functional characterisation of the majority of these candidates is lacking. Our model, the anthozoan *Nematostella vectensis*, is a sea anemone with a sequenced genome and a mode of embryonic development that is amenable to gene manipulation experiments (Putnam et al., 2007; Technau and Steele, 2011). To date, analyses of the cellular context of neurogenesis in *Nematostella* have revealed a number of similarities to bilaterians; most strikingly, the generation of neurons from neural progenitor cells (NPCs) which lie within epithelial layers (Nakanishi et al., 2012; Richards and Rentzsch, 2014). These features set *Nematostella* apart from the other most-studied cnidarian, the hydrozoan *Hydra*, which generates neurons from endodermally derived stem cells that also possess broader non-neural developmental potential (Bosch and David, 1987).

Neurogenesis in bilaterians is regulated by conserved signalling molecules and transcription factors, with Notch signalling, a subset of Group A basic helix-loop-helix (bHLH) (so-called ‘proneural’ genes), and SoxB genes being central elements of neurogenic networks. Despite a common involvement in early neurogenesis, functional studies addressing the contributions and interactions of these components in vertebrates (mouse, chicken, frog and zebrafish) and the fruit fly *Drosophila melanogaster* have revealed that their roles and interactions can differ significantly between species (Bertrand et al., 2002; Ernsberger, 2015; Hartenstein and Wodarz, 2013; Imayoshi and Kageyama, 2014; Louvi and Artavanis-Tsakonas, 2006; Ninkovic and Gotz, 2014; Pierfelice et al., 2011; Reiprich and Wegner, 2015). In *Nematostella*, previous works have also proposed roles for the Notch pathway, SoxB and bHLH genes in the regulation of various aspects of neural development, suggesting that these factors have underpinned neurogenesis in the most recent eumetazoan ancestor (Layden et al., 2012; Layden and Martindale, 2014; Marlow et al., 2012; Richards and Rentzsch, 2014; Watanabe et al., 2014). Here, we examine the expression and interactions of Notch, SoxB and bHLH genes during early ectodermal neurogenesis in *Nematostella* in order to gain a more integrated understanding of primary neurogenic events in this species, and to provide fresh insight into conserved and divergent aspects of eumetazoan neurogenesis.

A key regulator of early neurogenesis in bilaterians is the Notch signalling pathway, which classically acts via a mechanism of lateral inhibition between neighbouring cells. In vertebrates, a central function of Notch signalling is to maintain NPCs in an undifferentiated state by inducing the expression of bHLH genes from the Hes family – which act as repressors of proneural bHLH genes (i.e. certain Group A bHLH genes belonging to the Atonal, Neurogenin and Achaete-scute families) [reviewed by Bertrand et al. (2002); Louvi and Artavanis-Tsakonas (2006)]. Upon downregulation of Notch signalling, proneural bHLH genes are relieved from this repression and can initiate a neural differentiation program. Concomitantly, proneural bHLH genes upregulate the expression of ligands for the Notch receptor, which leads to an activation of Notch signalling in neighbouring cells, causing them to remain in a neural progenitor state. In *Drosophila*, the regulatory relationships between Notch, Hes and proneural bHLH genes are highly similar; however, Notch signalling in *Drosophila* acts in the selection of neural progenitor cells from ectodermal cells, rather than in the maintenance of an undifferentiated population of NPCs (Hartenstein and Wodarz, 2013). Moreover, proneural bHLH genes from different subfamilies have predominant roles in the generation of neural progenitor subtypes [e.g. Fode et al. (2000); Jarman et al. (1994); Sommer et al. (1996)]. Notwithstanding these differences, inactivation of Notch signalling results in a ‘neurogenic’ phenotype in both vertebrates and *Drosophila*, i.e. the generation of an excess of neurons [e.g. Chitnis et al. (1995); Lehmann et al. (1983)].

Similarly, in *Nematostella*, inhibition of Notch signalling via treatment with the γ-secretase inhibitor DAPT causes an increase in neurogenic gene expression and a downregulation of *Hes* genes (Layden and Martindale, 2014; Marlow et al., 2012). However, whereas the numbers of neurons are increased, the population of a second major class of neural cells, the nematocytes, is diminished (Marlow et al., 2012). This suggests that in *Nematostella*, unlike in bilaterians, either the primary function of Notch is to regulate a decision making process between alternate neural fates, or that Notch has different roles in different neural cell types (Marlow et al., 2012). Further evidence of dissimilarity between bilaterian and *Nematostella* Notch signalling was found in a recent study, which was unable to link the expression and function of *Hes* genes, or of the canonical Notch co-factor *suppressor of Hairless*, to the activity of Notch during *Nematostella* neural development (Layden and Martindale, 2014). As such, even though a role for Notch-Delta signalling in regulating eumetazoan neurogenesis is broadly conserved, there appears to be significant variation in the modes of deployment of this pathway between species. Indeed, analyses of Notch signalling in the *Hydra* nervous system have found that Notch does not restrict neurogenesis, instead playing a role in the later differentiation processes of nematocytes (Käsbauer et al., 2007).

Acting alongside Notch signalling in bilaterians, SoxB genes are a subfamily of the HMG-box domain-containing Sox transcription factor family, the activities of which often concern the maintenance of stem cell identity versus regulated cellular differentiation [reviewed by Kiefer (2007)]. Diverse bilaterians express *SoxB* orthologues during early neurogenesis [e.g. Kerner et al. (2009); Lowe et al. (2003); Pioro and Stollewerk (2006); Uy et al. (2012)], and functional studies in *Drosophila* and chick have identified roles for *SoxB* genes in both in the promotion and maintenance of NPCs, as well as in the induction of neural differentiation [e.g. Bylund et al. (2003); Overton et al. (2002)]. In the hydrozoan *Clytia hemisphaerica*, five *SoxB* orthologues have been described; their expression is associated with both stem and differentiated cells of the nervous system, but there has been no functional characterisation of these genes (Jager et al., 2011). The expression of *SoxB* orthologues has also been linked to early neurogenesis in *Nematostella*, with multiple representatives of the SoxB family being expressed either in broad patterns or localised to single cells, in the ectoderm and endoderm prior to the overt differentiation of neurons in these layers (Magie et al., 2005). We previously have reported the expression and function of one of these genes, *NvSoxB(2)*, which we found to be localised to proliferating NPCs and required for the proper generation of the sensory and ganglion neurons and nematocytes that comprise the larval nervous system (Richards and Rentzsch, 2014). Another orthologue, *NvSox1*/*NvSoxB2a* has recently been shown to be important specifically for the development of the oral nervous system in *Nematostella* (Watanabe et al., 2014).

In the current study, we demonstrate that Notch signalling in *Nematostella* negatively regulates the numbers of both neurons and nematocytes, and thus acts in a manner more akin to the bilaterian condition than previously recognised. We identify *NvAth-like* (Atonal/Neurogenin family) as an early-acting, broadly expressed bHLH gene that is co-expressed with *NvSoxB(2)* in dividing neural progenitor cells and required for proper nervous system development. Despite this co-localisation, the initial expression of *NvAth-like* and *NvSoxB(2)* appears to be independent from the other, suggesting that the mechanisms by which these genes promote neurogenesis are distinct. We find that *NvAth-like*, *NvSoxB(2)* and *NvAshA* demonstrate different temporal sensitivity to Notch inhibition, yet, notably, we did not find evidence of a classical lateral inhibitory mechanism underpinning the scattered patterning of neural progenitors in *Nematostella*. Finally, we determine that even in the absence of Notch inhibition, neurogenesis does not persist in embryos lacking NvSoxB(2).

## RESULTS

### DAPT causes an increase in the number of neurons and nematocytes generated in *Nematostella*

**Fig. 1.**
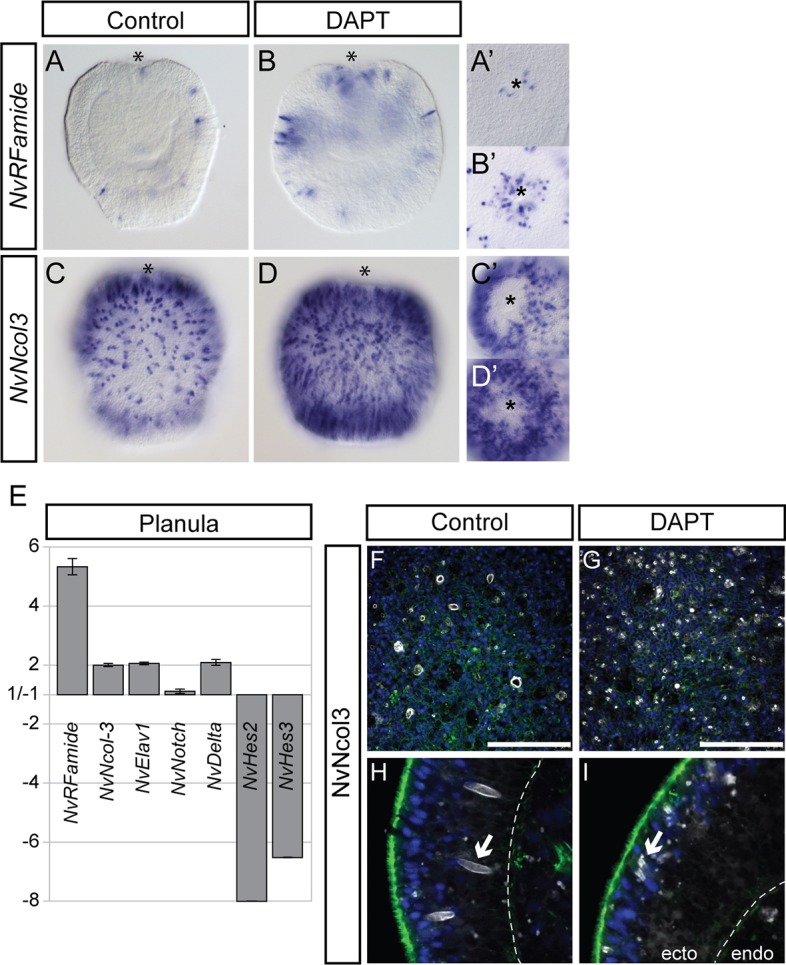
**Treatment with DAPT increases the numbers of neurons and nematocytes, but inhibits nematocyte differentiation.** (A-D′) Planula larvae raised in DAPT show an increase in cells expressing the neuron marker *NvRFamide* (A,B) and the nematocyte marker *NvNcol3* (C,D); this is particularly evident at the oral pole (A′,B′,C′,D′). (E) RT-qPCR confirms the increase of neural gene expression resulting from DAPT treatment; *NvDelta* is also increased, whereas *NvHes2* and *NvHes3* are downregulated. Graphs show the mean and s.e. of the relative fold change in expression between DAPT and DMSO (control) treatments across three biological replicates (values between 1.0 and −1.0 are omitted, as they indicate no change). An increase in nematocyte number is seen when immunostaining using anti-NvNcol3 (white) (F,G); however, the nematocysts within these cells are improperly formed (compare arrows in H,I). (A,B) Optical mediolateral sections of WMISH; (A′,B′,C′,D′) oral surface views; (C,D) surface views. (F,G) Confocal scan of lateral surface. (H,I) Confocal cross-section within lateral ectoderm. Asterisk, oral pole. Blue, DAPI; green, phalloidin; dashed line demarcates ectoderm and endoderm. Scale bars: 50 µm.

Previous studies in *Nematostella* reported an increase in the expression of marker genes for sensory and ganglion cells after treatment with DAPT [a chemical inhibitor of γ-secretase (Micchelli et al., 2003)], and tied this effect to an inhibition of Notch signalling activity in DAPT-treated embryos (Layden and Martindale, 2014; Marlow et al., 2012). We confirmed the downregulation of *NvHes* genes, and the upregulation of *NvDelta* and the neural markers *anthoRFamide* (*NvRFamide*) and *NvElav1* (Marlow et al., 2009) after DAPT treatment via RT-qPCR and *in situ* hybridisation (Fig. 1A,B,E; supplementary material Fig. S1). It was also previously reported that DAPT treatment decreases the number of nematocytes (Marlow et al., 2012); however, in contrast to this, we observed an increase in the expression of the pan-nematocyte marker *NvNcol3* (Zenkert et al., 2011) (Fig. 1C,D). To resolve these observations, we investigated the downstream effects of DAPT-induced expression changes on the mature neural populations of planula larvae using antibody staining. We found that, in accordance with the increase in *NvElav* mRNA expression, DAPT-treated *NvElav1::mOrange* transgenic larvae (Nakanishi et al., 2012) displayed an increased number of mOrange^+^ sensory cells with mature morphology (i.e. sensory cilium, basal neurites) (supplementary material Fig. S1). However, anti-Ncol3 immunolabelling (Zenkert et al., 2011) revealed that the differentiation of nematocytes in DAPT-treated larvae was severely perturbed (Fig. 1F-I). Whereas the number of immunoreactive cells is increased in treated samples, NvCol3 was no longer localised to oval-shaped capsules within each developing nematocyte. Rather, NvCol3 was distributed in numerous small foci throughout the cells, suggesting that treatment with DAPT disrupts the formation of nematocysts, the proteinaceous capsules that are definitive of nematocytes.

To confirm that the effects we observed from DAPT treatment were specific to an inhibition of Notch signalling, we also knocked down *NvNotch* using a splice-blocking morpholino (Layden and Martindale, 2014). We found that the pattern of gene expression changes in morphants were the same as in DAPT-treated animals, although at a lesser magnitude of change (supplementary material Fig. S1). We infer that this difference might reflect a reduced persistence of the morpholino knockdown treatments (injected into zygotes), when compared with the continuous exposure to DAPT experienced in the drug inhibition experiments.

### *NvAshA* and *NvAth-like* co-localise with subsets of *NvSoxB(2)*-expressing cells

To integrate the roles of *NvSoxB(2)* as a positive regulator of neural progenitor cells and Notch signalling as a restrictive signal on neural development, we examined the relationship between *NvSoxB(2)* and Group A bHLH genes during *Nematostella* neurogenesis. *Nematostella* has four Achaete-scute family genes (Simionato et al., 2007), but only one of these, *NvAshA*, is expressed in a scattered pattern in early neurogenesis (Layden and Martindale, 2014). However, as the expression of *NvAshA* begins later and is more restricted than *NvSoxB(2)*, and as the activity of *NvAshA* is limited to a subset of neural cell types (Layden et al., 2012; Layden and Martindale, 2014), we were interested to identify an *Atonal*/*Neurogenin* orthologue that might play an earlier and potentially broader role than *NvAshA*. We focused on *NvAth-like* (Layden et al., 2012; Layden and Martindale, 2014; Marlow et al., 2012), which was initially published as *Nem10* (Simionato et al., 2007). While there are a number of potential *Atonal*/*Neurogenin* orthologues in *Nematostella*, in phylogenetic analyses NvAth-like tends to associate most closely with NeuroD and Neurogenin proteins, as an outgroup to these families (Simionato et al., 2007). A direct comparison of the bHLH domains of closely related *Nematostella* Group A bHLH proteins and those of representative bilaterians also indicates that NvAth-like is most similar to the NeuroD and Neurogenin families (supplementary material Fig. S2).

**Fig. 2.**
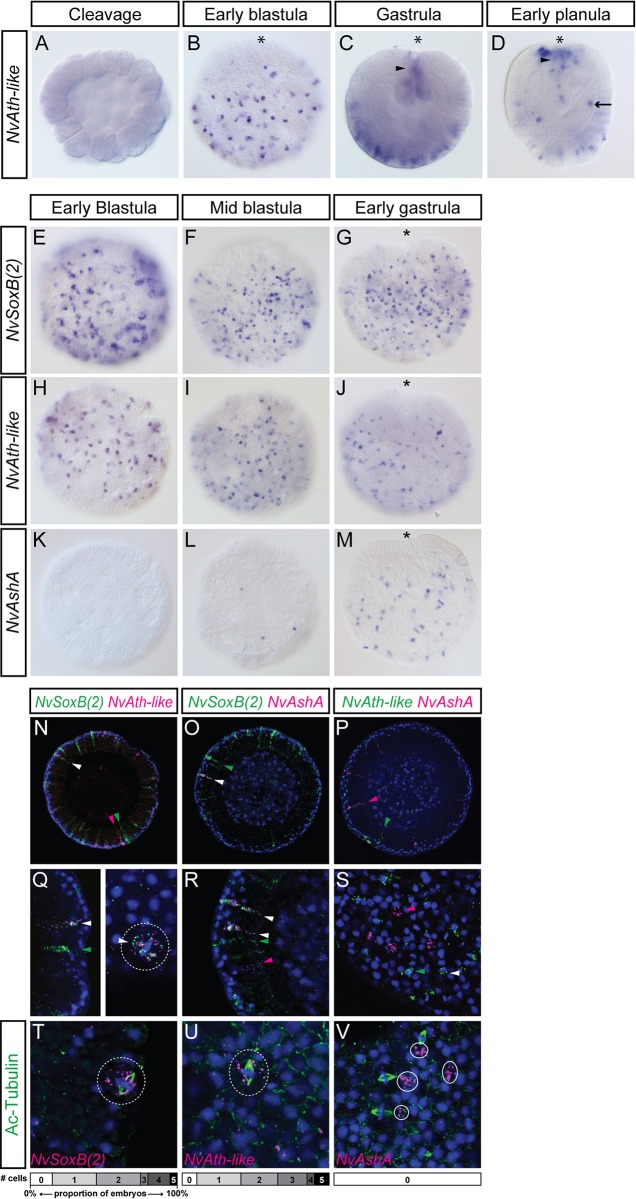
***NvSoxB(2)*, *NvAshA* and *NvAth-like* are co-expressed during development, but *NvAshA* is not found in mitotic cells.** (A-D) Expression of *NvAth-like* is first detected in early blastulae, in scattered cells that are absent from one region of the embryo (presumptive endodermal plate) (B). In gastrulae (C), expression is in scattered ectodermal cells predominantly in the aboral region of embryos, also in the developing pharynx (arrowhead). These domains are similar in early planulae (D), with the addition of newly expressing cells in the endoderm (arrow). Whereas *NvSoxB(2)* (E-G) and *NvAth-like* (H-J) are already expressed in early blastula stages, *NvAshA* (K-M) is expressed in only a few cells by mid-blastula stage, and is not broadly expressed until the early gastrula. (A,C,D) Mediolateral optical sections; (B,E-M) surface views. (N-V) Double FISH at blastula and gastrula stages shows some co-localisation of *NvSoxB(2)* with both *NvAth-like* and *NvAshA* (N,O,Q,R), but minimal co-localisation between *NvAth-like* and *NvAshA* (P,S). Based on the elongated shape of the DAPI nuclear staining, mitotic cells expressing both *NvSoxB(2)* and *NvAth-like* were identified (dashed circle in Q). By staining spindles with an acetylated tubulin antibody (green), we observed mitotic cells expressing *NvSoxB(2)* (T) and *NvAth-like* (U) in single FISH (dashed circles). *NvAshA* transcripts (circled in V) were never found in cells undergoing mitosis. Charts show how many mitotic, gene-expressing cells were observed in each of 20 early- and mid-gastrula embryos/gene. White arrowheads, co-localisation; green and pink arrowheads, single transcript localisation. Blue, DAPI; Ac-Tubulin, anti-acetylated tubulin. Asterisk, oral pole.

During development, *NvAth-like* expression is first detected post-cleavage, in scattered cells of the hollow blastula; these cells are not found on one pole of the embryo, presumably the forming pre-endodermal plate (Fig. 2A,B). In gastrulae, *NvAth-like* is detected in scattered ectodermal cells, more predominantly in the aboral two-thirds of the embryo, as well as in the invaginating pharyngeal ectoderm (Fig. 2C). By early planula stage, expression is additionally detected in scattered cells of the endoderm (Fig. 2D). *NvAth-like* is thus expressed in a manner very similar to other *Nematostella* transcription factors with roles in early ectodermal nervous system development [see e.g. Layden et al. (2012); Richards and Rentzsch (2014)]. Comparatively, *NvAth-like* expression is initially detected at the same timepoint as *NvSoxB(2)*, in the early blastula, while *NvAshA* expression is first detected considerably later, in the early gastrula (Fig. 2E-M).

In contrast to these data, an alternative expression pattern for *NvAth-like* was recently published (Watanabe et al., 2014), in which *NvAth-like* (renamed as *NvArp3*) was reported to be expressed in a broad oral domain during blastula and gastrula stages. To clarify this disparity we generated a probe from the *NvArp3* clone provided by Watanabe and colleagues. We detected the *NvArp3* probe in scattered cells throughout early embryos, confirming our description of *NvAth-like* expression (supplementary material Fig. S3). We saw no evidence to support *NvAth-like*/*NvArp3* being expressed in a broad oral domain.

We next undertook co-expression studies to investigate whether *NvAth-like*, *NvSoxB(2)* and *NvAshA* are expressed in the same cells and might thus regulate similar events during the development of neural cell types. We found that *NvAth-like* is expressed in a subset of *NvSoxB(2)*-expressing cells at late blastula and early gastrula stage, including some dividing cells (Fig. 2N,Q). In addition to these co-expressing cells, both genes were also detected in single-labelled cells (Fig. 2N,Q). The same situation was observed for *NvSoxB(2)* and *NvAshA*; however, the overlap in expression was smaller and we did not detect any dividing co-expressing cells (Fig. 2O,R). By contrast, we found very minimal overlap between *NvAth-like* and *NvAshA* (Fig. 2P,S). By immunolabelling mitotic spindles with anti-acetylated Tubulin, we confirmed that both *NvSoxB(2)* and *NvAth-like* can be expressed in dividing cells, whereas *NvAshA* could not (Fig. 2T-V). We found similar patterns of expression in the mid-body ectoderm and pharyngeal ectoderm of early planula larvae, including the co-localisation of *NvAth-like* and *NvSoxB(2)* in dividing cells (supplementary material Fig. S4). In the endoderm, however, while *NvSoxB(2)* and *NvAth-like* are co-expressed in some cells, *NvSoxB(2)* is expressed in many more cells than *NvAth-like*, and *NvAshA* is not expressed at all in the endoderm at this stage (supplementary material Fig. S4). From these data, a possible model of the relationship between these three genes during early *Nematostella* neurogenesis begins to emerge – *NvAth-like* and *NvSoxB(2)* are expressed in dividing cells [probably neural progenitors, see Richards and Rentzsch (2014)], with *NvAshA* being activated at a later point in the development of these cells – while they are still expressing *NvSoxB(2)*, but after *NvAth-like* expression has mostly ceased.

### *NvAth-like* is required for the development of neurons and nematocytes

**Fig. 3.**
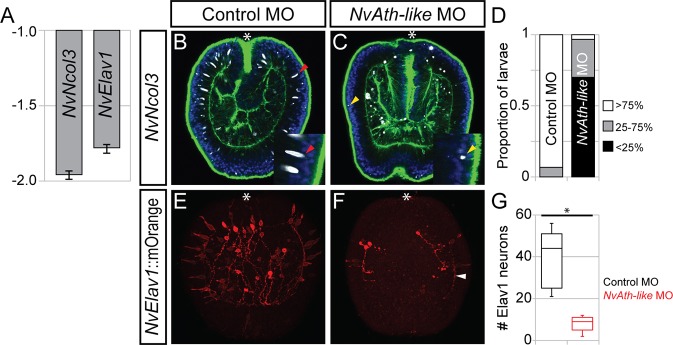
***NvAth-like* is a positive regulator of neurogenesis.** (A) Morpholino inhibition of *NvAth-like* reduces the expression of the nematocyte marker *NvMinicollagen3* (*NvNcol3*) and the neuron marker *NvElav1* at planula stage two- and 1.75-fold, respectively. Graph depicts means and s.e. of relative fold changes in expression between *NvAth-like* MO- and control MO-injected embryos across three biological replicates (values between 1.0 and −1.0 are omitted, as they indicate no change). (B,C) Anti-Ncol3 immunostaining (white) reveals a lack of nematocyst capsules (red arrowheads) in *NvAth-like* morphants; some small spots of anti-Ncol3 staining are present in the apical part of the ectoderm (yellow arrowheads). (D) Scored phenotypes of injected embryos, categories indicate an estimation of the proportion of nematocysts relative to WT; *n*=30 embryos/morpholino. (E,F) *NvAth-like* morphants have reduced numbers of *NvElav1*::mOrange^+^ neurons (red) at mid-planula stage (F), compared with control-injected animals (E); the polarity of neurite growth (e.g. white arrowhead) is mostly maintained. (G) Box-and-whisker plot confirming the significant (*) decrease in *NvElav1*::mOrange^+^ neurons in *NvAth-like* morphants (*P*=4.1E-08); *n*=12 planulae/condition. Whiskers depict 1.5IQR above and below the third and first quartile, respectively. (B,C) ICC, mediolateral confocal sections; blue, DAPI; green, phalloidin. (E,F) ICC, maximum projections from surface to larval centre. Asterisk (B,C,E,F) marks the oral pole.

As the partial co-expression of *NvAth-like* with *NvSoxB(2)* suggests a role in neural progenitor cell regulation, we next investigated whether blocking the translation of *NvAth-like* via the injection of morpholino oligonucleotides ‘*NvAth-like* MO’ (see supplementary material methods for morpholino sequences and Fig. S5 for control experiments) would provoke a change in the expression of neural differentiation markers. At planula stage, we found that the expression of the nematocyte marker *NvNcol3* (Zenkert et al., 2011) was reduced twofold in *NvAth-like* morphants compared with control MO-injected samples, whereas the sensory and ganglion cell marker *NvElav1* was reduced 1.75-fold (Fig. 3A). To identify what effect this change in gene expression has on the morphology of the planula nervous system, we first used an antibody against NvNcol3 (Zenkert et al., 2011) and observed far fewer nematocysts in *NvAth-like* morphants (Fig. 3B-D). By injecting *NvAth-like* MO into the *NvElav1::mOrange* transgenic line, we then confirmed that NvAth-like is also required for the proper development of Elav1^+^ sensory cells and ganglion neurons (Fig. 3E-G). Of the few neural cells that did develop in *NvAth-like* morphants, the majority still exhibited the aboral orientation of neurite outgrowth that characterises the Elav1^+^ nervous system at this stage (Fig. 3F) (Nakanishi et al., 2012).

### DAPT-induced changes in the expression of *NvAshA*, *NvAth-like* and *NvSoxB(2)* arise at different development timepoints

**Fig. 4.**
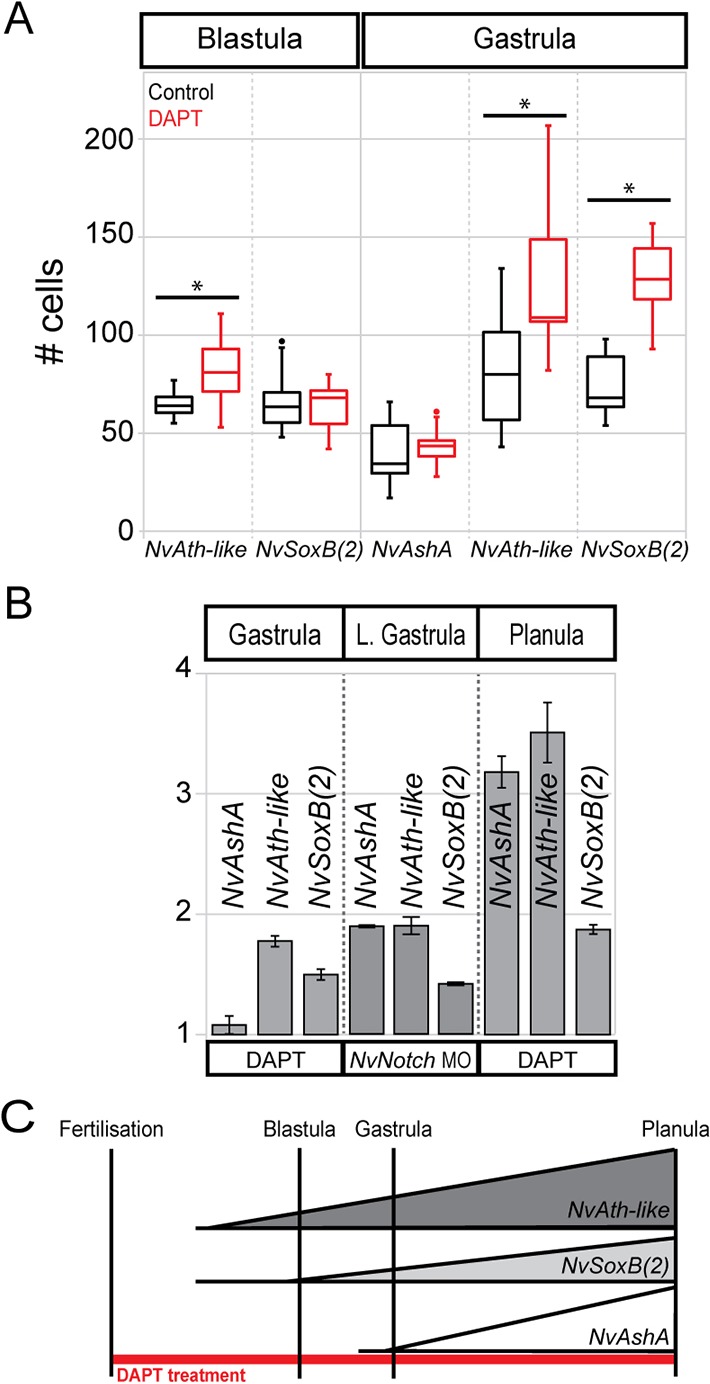
**DAPT causes a differential increase in the expression of *NvAshA*, *NvAth-like* and *NvSoxB(2)* over developmental time.** (A) Box-and-whisker plot showing the number of cells expressing each gene counted in a defined section of ectoderm, *n*=10 embryos/treatment/stage. Significant increases (*) in cell number were observed at both blastula (*P*=0.007) and gastrula (*P*=0.001) stage for *NvAth-like* and at gastrula stage for *NvSoxB(2)* (*P*=3.8E-06). Whiskers depict 1.5IQR above and below the third and first quartile, respectively. (B) RT-qPCR shows a similar response to DAPT at the transcript level, with all genes being upregulated at both the gastrula and planula stages, but with the response of *NvAshA* at gastrula stage being very mild. At late gastrula stage, morpholino knockdown of *NvNotch* causes an increase in target gene expression which correlates with the expression changes recorded via DAPT inhibition. Graph depicts means and s.e. of relative fold changes in expression between treatment and control samples across three biological replicates (values between 1.0 and −1.0 are omitted, as they indicate no change). (C) Summary of timing and magnitude of gene expression changes as a result of DAPT treatment in *Nematostella*.

Previous works have shown that the expression levels of *NvSoxB(2)*, *NvAth-like* and *NvAshA* are sensitive to the inhibition of Notch signalling by DAPT at late gastrula stage [ca. 30 h post-fertilisation (hpf)] (Layden and Martindale, 2014) and at mid-late planula stage (ca. 85 hpf) [*NvAth-like* and *NvAshA*, see Marlow et al., 2012)]. However, as we have identified the onset of *NvAth-like* and *NvSoxB(2)* expression in early blastulae (10 hpf), and of *NvAshA* in late blastulae (18 hpf), we hypothesised that these genes are already regulated by Notch signalling at earlier developmental stages, during neurogenic events that occur before the onset of neural differentiation in late gastrula/early planula. Accordingly, we examined early changes in spatial expression (at 16 hpf and 24 hpf via *in situ* hybridisation) and later changes in transcript abundance (at 24 hpf and 48 hpf via RT-qPCR) of all three genes in embryos treated with DAPT. From this, we determined that *NvAth-like* is the first gene to show a response to DAPT treatment, with a significant change in the number of *NvAth-like*-expressing cells being recorded at blastula stage (16 hpf) (Fig. 4A). At this time, there was no change in the number of cells expressing *NvSoxB(2)*, and *NvAshA* expression was undetectable (Fig. 4A). By gastrula stage (24 hpf), both *NvAth-like* and *NvSoxB(2)* showed a significant response to DAPT treatment, whereas *NvAshA* remained unchanged (Fig. 4A) (for representative micrographs used for scoring cell numbers see supplementary material Fig. S6). Regarding transcript abundance, the data at gastrula stage confirm the pattern seen when scoring cellular expression – in that the response of *NvAth-like* and *NvSoxB(2)* is stronger than that of *NvAshA*. By planula stage (48 hpf), all genes are upregulated by DAPT treatment, with *NvAth-like* (3.5-fold) and *NvAshA* (threefold) showing a greater response than *NvSoxB(2)* (twofold) (Fig. 4B). To confirm that these effects relate to an inhibition of Notch signalling, we performed morpholino knockdown of *NvNotch* and quantified gene expression changes at an intermediary stage – the late gastrula. Compared with the DAPT-treated gastrula, we saw a similar upregulation of *NvAth-like* and *NvSoxB(2)* in late gastrula-stage *NvNotch* morphants (Fig. 4B). Notably, we were also able to identify the onset of *NvAshA* upregulation at this late gastrula timepoint (Fig. 4B). Consistent with these data, we did not register any significant upregulation of neural differentiation markers (*NvRFamide*, *NvNcol3* or *NvElav1*) before early planula stage (48 hpf) [Fig. 1; supplementary material Fig. S1; see also Marlow et al. (2012)].

### Reciprocal morpholino knockdowns reveal interactions between *NvSoxB(2)*, *NvAshA* and *NvAth-like*, but demonstrate little cross-talk with the Notch pathway

**Fig. 5.**
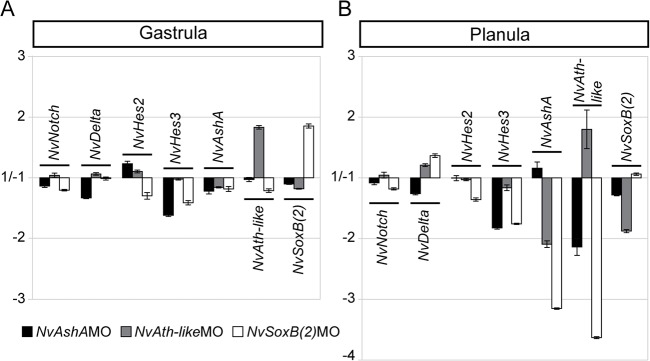
***NvSoxB(2)*, *NvAshA* and *NvAth-like* reciprocally regulate, but have minimal impact on Notch components.** The changes in gene expression that occur in gastrulae (A) and planulae (B) as a result of *NvSoxB(2)*, *NvAshA* and *NvAth-like* knockdown. Graphs show means and s.e. of relative fold changes in expression between control MO and gene-specific MO-injected embryos across three biological replicates (values between 1.0 and −1.0 are omitted, as they indicate no change).

To investigate the functional interactions between *NvSoxB(2)*, *NvAshA*, *NvAth-like* and Notch pathway components, we carried out a series of reciprocal morpholino knockdown experiments. These experiments demonstrated that, at gastrula stage, the effects of each morpholino are minor, with only the self-repression of *NvAth-like* and *NvSoxB(2)*, and a downregulation of *NvHes3* in *NvAshA* morphants showing a marked response. However, by planula stage, *NvAth-like* and *NvAshA* are downregulated in *NvSoxB(2)* morphants; *NvAshA* and *NvSoxB(2)* are downregulated in *NvAth-like* morphants, and *NvAth-like* is downregulated in *NvAshA* morphants (Fig. 5). *NvHes3* remains downregulated in *NvAshA* and now also in *NvSoxB(2)* morphants, but there is still negligible effect on the expression of *NvNotch*, *NvDelta* or *NvHes2* in any of the morpholino-injected samples. Combined, these data show that *NvSoxB(2)* acts upstream of *NvAshA*, and they reveal the existence of co-dependencies between *NvSoxB(2)* and *NvAth-like*, and between *NvAshA* and *NvAth-like* at planula stage. Despite *NvAth-like* and *NvSoxB(2)* being, at least partially, co-expressed since early blastula, we find little regulatory interaction between these genes in pre-planula stages. Moreover, we find that there is minimal feedback of *NvSoxB(2)*, *NvAshA* and *NvAth-like* onto Notch components and *Hes* family genes.

These experiments also show self-regulated repression of *NvSoxB(2)* (gastrula stage) and *NvAth-like* (gastrula and planula stages); however, an increase in target transcripts (due to mRNA persistence or compensation by the embryo) has been discussed as a possible artefact in morpholino knockdown experiments (Eisen and Smith, 2008). As such, even though we find the potential of these interactions intriguing, we hesitate to draw strict conclusions from these data at present.

### Inducing hyperneurogenesis with DAPT does not rescue the inhibition of nervous system development caused by *NvSoxB(2)* knockdown

**Fig. 6.**
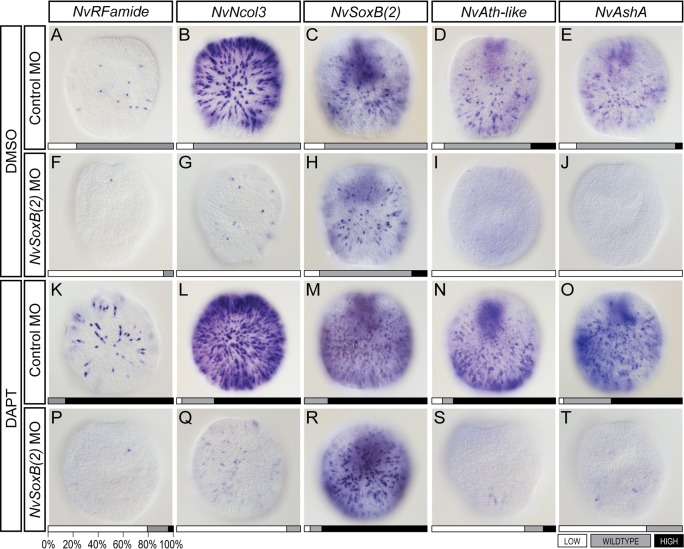
**Blocking Notch signalling with DAPT does not rescue the inhibition of neural development caused by *NvSoxB(2)* knockdown.** Embryos injected with control MO and raised in DMSO showed expected wild-type gene expression at early planula stage (A-E), whereas those injected with *NvSoxB(2)* MO and raised in DMSO displayed a reduction in the expression of neural markers *NvRFamide*, *NvNcol3* and the bHLH genes *NvAth-like* and *NvAshA* (F,G,I,J). *NvSoxB(2)* was expressed as normal (H). Expression of all genes was expanded in embryos injected with control MO and raised in DAPT (K-O). By contrast, DAPT treatment of *NvSoxB(2)* MO-injected embryos resulted in a reduction of all genes, except for *NvSoxB(2)* (R). Larvae were scored as displaying wild-type (WT), diminished (LOW) or expanded (HIGH) levels of gene expression. Scores are shown on the bottom of each panel as a proportion of 100%. A minimum of 20 larvae were examined for each gene/treatment. All panels show superficial lateral views of early planula larvae, with the oral pole to the top.

As *NvSoxB(2)* knockdown inhibits neurogenesis, and Notch inhibition promotes neurogenesis, we wanted to test whether these opposing activities had an epistatic relationship. To do so, we performed double-knockdown experiments in which *NvSoxB(2)* MO-injected embryos were raised in DAPT. We analysed the outcome from this experiment via *in situ* hybridisation at early planula stage. As expected, we recorded a lack of neurogenesis in *NvSoxB(2)* morphants (Fig. 6F-J), including the downregulation of *NvAth-like* and *NvAshA*. Also similar to expectations, we observed expanded expression patterns in DAPT-treated animals, with the number of cells expressing each gene being increased (Fig. 6K-O). When we examined the double-treated samples, we found that animals injected with *NvSoxB(2)* MO no longer exhibited a hyperneurogenic phenotype when raised in DAPT (Fig. 6P-T). Rather, the observed phenotypes in the double treatment resembled that seen in *NvSoxB(2)* single knockdowns. The exception to this pattern being *NvSoxB(2)* itself, which maintained an expanded expression pattern in the double treatment. Thus, we find that knockdown of *NvSoxB(2)* causes a reduction in neural gene expression, including that of the Group A bHLH genes *NvAth-like* and *NvAshA*, despite the repression of Notch-mediated negative regulation of these genes.

## DISCUSSION

Our results support a model in which Notch signalling negatively regulates *Nematostella* neurogenesis by limiting the number of *NvAth-like* expressing neural progenitor cells. *NvSoxB(2)* is also expressed in these cells, but this expression appears to be independent of *NvAth-like* and might occur at a later point in the developmental program of the progenitor population. Both genes are required for proper nervous system development, with the knockdown of either causing a loss of differentiated neural cells. Notably, the requirement for *NvSoxB(2)* is also independent of Notch signalling; embryos in which Notch is inhibited do not develop nervous systems in the absence of *NvSoxB(2)*. Together, we find that the roles of Notch, SoxB and bHLH genes in *Nematostella* are broadly conserved with their roles in bilaterians; however, the evidence for NvNotch signalling via a canonical Notch mechanism remains ambiguous.

### Notch signalling negatively regulates neurogenesis in *Nematostella*

The role of Notch signalling in *Nematostella* appears very similar to bilaterian systems. Inhibition of Notch via DAPT or *NvNotch* knockdown leads to the upregulation of the Group A bHLH genes *NvAth-like* and *NvAshA*, and, consequently, the numbers of both neurons and nematocytes are increased causing a classical ‘neurogenic’ phenotype. Our results differ from a previous study, which reported that DAPT caused an increased number of neurons but a reduced number of nematocytes in *Nematostella* (Marlow et al., 2012). The contradiction between our data and those of Marlow et al. (2012) is probably due to the different methods used to assess the nematocyte population. Marlow and colleagues used DAPI staining, which only detects a subset of mature nematocytes (Szczepanek et al., 2002), and thus, they were unable to detect the excess of immature nematocytes that arises in DAPT-treated larvae. By examining *NvNcol3* gene expression and the localisation of an antibody against NvNcol3, we could observe that the population of nematocytes is increased, while the proper differentiation of nematocysts is perturbed. Notably, a similar DAPT-induced inhibition of nematocyst maturation has been described in the hydrozoan *Hydra* (Käsbauer et al., 2007), suggesting a conserved role for Notch signalling in promoting nematocyst differentiation in cnidarians. However, as this phenotype has currently only been observed via DAPT treatment, the recapitulation of this effect using independent means of Notch manipulation will be required to confirm this novel hypothesis.

Although our data suggest a typical anti-neurogenic function of Notch signalling in *Nematostella*, it remains to be shown whether Notch functions in the maintenance of a pool of neural stem cells and the regulation of the timing of their differentiation (as in vertebrates), in the restriction of NPC fate acquisition within groups of equivalently poised ectodermal cells (as in *Drosophila*), or, indeed, in a novel manner that is distinct to the Cnidaria/Anthozoa. In support of this third possibility, a recent study concluded that Notch in *Nematostella* regulates neural differentiation, and does so via a non-canonical (*Hes* and *suppressor of Hairless* independent) mechanism (Layden and Martindale, 2014). Favouring this hypothesis, we did not observe classical feedback regulation of *NvDelta*, *NvNotch* or *NvHes* genes in response to *NvAth-like* or *NvAshA* knockdown (although there is an increase in *NvDelta* expression after DAPT treatment, as would be expected were Notch repressing the ability of cells to signal via *Delta* ligands). Furthermore, in contrast to Layden and Martindale (2014), we recorded a three- to fourfold reduction in *NvHes* gene expression after *NvNotch* morpholino knockdown (supplementary material Fig. S1). This suggests that *NvHes2* and *-3* are indeed targets of *NvNotch*, and thus, that Notch signalling in *Nematostella* neurogenesis can occur via a canonical Hes-dependent mechanism. However, as overexpression of *NvHes2* and *-3* shows no impact on neural gene expression, and overexpression of *NvDelta* or the *NvNotch* intracellular domains does not affect *NvHes* expression (Layden and Martindale, 2014), current data do not allow for a definitive conclusion on the mechanism of signalling in *Nematostella* neurogenesis. In addition, we frequently find that the expression changes recorded from DAPT treatments are of greater magnitude than those obtained via *NvNotch* morpholino knockdown or other methods of manipulation (Layden and Martindale, 2014; Marlow et al., 2012). In future studies, it will be important to determine whether this disparity stems solely from the technical difference between prolonged drug treatment versus microinjection into zygotes, or whether DAPT has additional effects on neurogenesis that are Notch independent.

At the level of spatial patterning, we do not see a striking change in the arrangement of cells expressing neural genes in Notch-inhibited animals. Classically, Notch acts to select cells from within a field of similar cells, and we do find that *NvAth-like* and *NvSoxB(2)* are expressed both in single cells and in pairs or small groups of cells during pre-larval stages. Notch inhibition does not lead to an obvious development of these ‘patches’ into differentiated neurons; however, the high variation in the spatial patterning of neural gene expression between animals of the same stage precludes definitive conclusions. We envisage that the refinement of hypotheses regarding Notch activity in *Nematostella* early neurogenesis will require an improvement in our ability to describe and distinguish cells belonging to specific sub-populations of neurons, and to identify neural cells at different stages of differentiation and cell cycling.

### Differential expression, regulation and interactions of *NvAth-like* and *NvAshA*

In bilaterians, group A bHLH genes from different subfamilies play distinct roles in the promotion of neuronal and glial cell subtypes, and can further act in regulatory cascades with early-expressed genes involved in fate speciﬁcation of neural progenitors and late-expressed genes regulating terminal differentiation [e.g. Bertrand et al. (2002); Cau et al. (1997); Lee (1997)]. When considering roles for *NvAth-like* and *NvAshA* in *Nematostella* neurogenesis, we note that the onset of expression and DAPT sensitivity of *NvAth-like* occurs earlier than that of *NvAshA*, and that *NvAth-like* is expressed in more cells, including cells undergoing mitosis. We also observed that *NvAth-like* and *NvAshA* transcripts are rarely found in the same cell; however, both are, at least partially, co-expressed with *NvSoxB(2).* Additionally, whereas *NvAshA* knockdown does not impact *NvSoxB(2)* expression, there is downregulation of *NvSoxB(2)* in *NvAth-like* morphants.

We consider two scenarios for these observations: first, that *NvAth-like* and *NvAshA* are both broadly expressed in a common neural population, but that they regulate early (*NvAth-like*) versus late (*NvAshA*) neurogenic activity, thus playing different roles during the progression of neural lineage development. As such, *NvAth-like* functions as a broadly acting proneural gene involved in the specification of NPCs, whereas *NvAshA* is involved in regulating differentiation programs during later stages of neurogenesis. Second, that *NvAth-like* and *NvAshA* promote different neural sub-types, which develop at different times – in this case, the bHLH genes might act within a combinatorial transcription factor code for neural cell identity in *Nematostella* [e.g. Guillemot (2007)]. In favour of the first scenario, *NvAshA* is downregulated after *NvAth-like* knockdown, suggesting that they are sequentially expressed within the same developing neural population. However, we also observe a downregulation of *NvHes3* after *NvAshA* knockdown that does not occur in *NvAth-like* morphants. Additionally, *NvAth-like* is downregulated in *NvAshA* morphants when assayed at planula stage – which would not be expected, were *NvAshA* to act strictly downstream of *NvAth-like* in all cell types. We consider it likely that these genes are flexibly deployed over the course of development, in multiple populations of neural sub-types, and that they might have different functions in cell types that develop at different stages. However, regarding the initial onset of neurogenesis in the ectoderm, we propose that *NvAth-like* is a key pro-neurogenic factor, due to its early expression in blastula stage embryos, its localisation in dividing (and thus pre-differentiated) cells, its early sensitivity to Notch signalling, and owing to the fact that knockdown of *NvAth-like* results in the development of fewer neural cells.

### Interplay between Notch, SoxB and bHLH genes in the regulation of neurogenesis in *Nematostella*

How Notch signalling, bHLH and SoxB genes interact to control the progression of neurogenesis from NPCs is only partially understood. For example, in chick, *SoxB1* genes can block the ability of proneural bHLH proteins to induce neurogenesis, independently of Notch-mediated transcriptional repression by *Hes* genes. In turn, proneural proteins can block *SoxB1* and induce *SoxB2* expression in order to promote neural differentiation (Bylund et al., 2003; Holmberg et al., 2008; Sandberg et al., 2005). In *Drosophila*, *SoxB* genes differentially regulate proneural genes of the Achaete-scute complex, depending on the domain of their expression (Buescher et al., 2002; Overton et al., 2002; Zhao and Skeath, 2002). However, despite being a positive regulator of proneural genes, *SoxNeuro* does not promote neuroblast formation by directly antagonising Notch signalling (Buescher et al., 2002). Similarly, the overexpression of *Sox3* in chick inhibits neural differentiation even in the absence of Notch activity (Holmberg et al., 2008).

We propose that this ‘parallel, yet interacting’ state of SoxB, Notch and bHLH gene regulation of NPCs, as described in chick and *Drosophila*, can also be applied to *Nematostella* neurogenesis. Specifically, we observe that the number of progenitor cells expressing *NvSoxB(2)* is negatively regulated by Notch activity; however, we find that *NvSoxB(2)* is required for neural development even when Notch signalling is inhibited and *Hes* repressors are downregulated. We also report that both *NvSoxB(2)* and *NvAth-like* are required for neural development and are co-expressed in early NPCs; however, knockdown of either gene does not affect the initial expression of the other. This suggests that *NvSoxB(2)* and *NvAth-like* promote neurogenesis via distinct mechanisms, or, alternatively, that their interaction occurs at the protein level [e.g. Bylund et al. (2003); Whittington et al. (2015)]. Based on bilaterian data, a possible Notch-independent role for *NvSoxB(2)* might include an interaction with TCF/β-catenin to modulate the activity of Wnt signalling (Agathocleous et al., 2009; Chao et al., 2007; Martinez-Morales et al., 2010; Overton et al., 2007). Certainly, expression data and overactivation experiments suggest that the Wnt pathway contributes to the regulation of NPCs in the *Nematostella* ectoderm (Kusserow et al., 2005; Marlow et al., 2013); but to date, Wnt function has only been studied in detail in the later-developing neural cells of the oral region (Watanabe et al., 2014).

At a broader scale, our results suggest that common principles of eumetazoan neurogenesis include complementary functions of *SoxB* genes and Notch signalling, acting as positive and negative regulators of neurogenesis, respectively, and the combinatorial deployment of Group A bHLH subfamilies during the specification and differentiation of diverse neural cell types [see also Fritzsch et al. (2015)]. Concerning cnidarian neurogenesis, areas of particular interest for future studies include the clarification of the Notch-Delta signalling mechanism, and the exploration of expansion and functional diversification within the SoxB and Group A bHLH gene families.

## MATERIALS AND METHODS

### *Nematostella* culture

Adults were cultured in 0.3× filtered seawater (NM) and induced to spawn as described in Fritzenwanker and Technau (2002). Egg packages were incubated for 25 min in 3% cysteine/NM after fertilisation to remove ‘jelly’. Embryos were raised in NM at 21°C. Staging scheme: early blastula, 12 h; blastula, 16 h; late blastula, 18 h; early gastrula, 20 h; gastrula, 24 h; late gastrula, 30 h; early planula, 48 h; mid planula, 72 h; late planula, 96 h.

### DAPT treatment and quantification

Fertilised eggs were de-jellied and then incubated in the dark in 10 µM DAPT/NM (Sigma) or 0.1% DMSO/NM (control). Solutions were changed every 12 h. To compare and quantify numbers expressing cells in control and DAPT-treated embryos, surface-level images were taken of ten embryos per stage. Cells were counted manually, assisted by Cell Counter (ImageJ). Prior to counting, image filenames were anonymised to avoid expectation bias. To assess whether the numbers of cells in the DAPT treatment were significantly changed from the control state, Student's *t*-test was applied (for unpaired samples with equal variance). Homoscedasticity was confirmed using Bartlett's test.

### Morpholino injection and quantification

Microinjection of morpholinos was carried out as described in Rentzsch et al. (2008). Fertilised eggs were injected with 250 μM-500 μM morpholino (Gene Tools) and 40 μg/ml Alexa Fluor488-conjugated Dextran (Invitrogen) in TAE buffer. Control injections were carried out using a generic control morpholino. For morpholino sequences and control experiments for *NvAth-like* MO, see supplementary material Table S2. The numbers of *NvElav1*::mOrange^+^ cells in *NvAth-like* morphants and controls (Fig. 1I,J) were manually counted in maximum projections from the surface to the centre of planulae. Counting and statistical assessment was carried out using the same methods as for DAPT experiments.

### Fluorescent/*in situ* hybridisation (FISH/ISH) and immunocytochemistry (ICC)

Experiments were conducted as described in Richards and Rentzsch (2014). For weak probes (requiring >48 h colour development), signal was enhanced using the TSA DNP system (PerkinElmer) following manufacturer's instructions. Details of probe sequences and antibody specifications/dilutions are provided in supplementary material methods and Tables S3 and S4. Specimens were imaged either on a Nikon Eclipse E800 compound microscope with a Nikon Digital Sight DSU3 camera or on a Leica SP5 confocal microscope. Figure plates were built using Adobe Design Standard CS5; images were cropped, and adjusted for brightness/contrast and colour balance; any adjustments were applied to the whole image, not parts.

### RT-qPCR

Quantification of gene expression changes was performed as in Richards and Rentzsch (2014). Primer pairs (see supplementary material Table S1) with PCR efficiencies of 90-105% were used for RT-qPCR, and two technical replicates were performed for each of the three biological replicates. Relative expression was calculated using the 2^−ΔΔCt^ method; control gene stabilities were assessed using RefFinder (http://fulxie.0fees.us/?type=reference), with *NvATPsynthase* and *NvELF1B* being selected as most stable for DAPT experiments, and *NvATPsynthase* and *NvRibPrL23* for morpholino experiments. In all graphs, the mean and s.e. of three biological replicates per experiment is presented; fold change values between 0 and 1/−1 are not shown, as they represent no change in expression.

### Transgenic animals

The *NvElav1*::mOrange embryos used in our experiments were derived from incrosses of the heterozygous stable transgenic line described in Nakanishi et al. (2012). In all images, expression of mOrange was visualised via immunostaining with anti-DsRed (rabbit, Clontech 632496; 1:100).
